# Intestinal/Peritoneal Tuberculosis in Children: An Analysis of Autopsy Cases

**DOI:** 10.1155/2012/230814

**Published:** 2012-12-19

**Authors:** Cecilia Ridaura-Sanz, Eduardo López-Corella, Ruy Lopez-Ridaura

**Affiliations:** Department of Pathology, National Institute of Pediatrics, 04530 Mexico City, DF, Mexico

## Abstract

Infection by *Mycobacterium bovis* is not infrequently identified in Mexico. Its relation to nonpasteurized milk products ingestion is well recognized with primary infection usually in the intestinal tract. The term “abdominal tuberculosis” includes peritoneal as well as primary and secondary intestinal tuberculosis. The clinical differentiation of these conditions is difficult. In this work, we reviewed the clinical and pathological features of 24 cases of children dying with tuberculosis in whom autopsy revealed abdominal disease in a referral hospital in Mexico City. We identified 8 cases of primary intestinal tuberculosis, with documentation of *M. bovis* in 6 of them, and 9 cases of secondary intestinal tuberculosis (primary pulmonary disease), all negative to *M. bovis*. Seven patients had peritoneal tuberculosis without intestinal lesions and with active pulmonary disease in 4 of them, and of the remaining three, two had mesenteric lymph node involvement suggesting healed intestinal disease. In this approach to abdominal tuberculosis, postmortem analysis was able to differentiate primary from secondary intestinal tuberculosis and to define the nature of peritoneal involvement. This discrimination gives rise to different diagnostic approaches and epidemiological and preventive actions, particularly in countries where tuberculosis is endemic and infection by *M. bovis* continues to be identified.

## 1. Introduction 

Tuberculosis continues to be a serious public health problem in Mexico, particularly for children. The incidence in 2007 was 13.49 cases/100 000 individuals and 5.077/100 000 in children under the age of 19 [1]. Around 16% of dairy cattle are infected with *M. bovis, *and 30% to 50% of their product is sold as nonpasteurized milk or cheese with a wide distribution [2]. Cheese consumption has been associated with cases of intestinal tuberculosis in Mexican children residing in New York City, and a similar situation was identified in Denver, Colorado, where most cases of tuberculosis caused by *M. bovis *occurred in Mexican residents [3, 4]. In a recent study in the vicinity of Mexico City, 13.8% of samples from symptomatic individuals with the tuberculosis complex corresponded by spoligotyping to *M. bovis *[2]. 

*M. bovis *is widely recognized as the causal agent of primary intestinal tuberculosis [5]. Tuberculosis infection in the abdominal cavity includes cases with involvement of the gastrointestinal tract, often with peritoneal extension, as well as cases of peritoneal infection without intestinal lesions. 

In a majority of publications, these conditions are grouped together under the designation of abdominal tuberculosis in view of a similar clinical expression and the difficult differential diagnosis. However, peritoneal tuberculosis and intestinal tuberculosis differ in etiology, pathogenesis, and, ideally, in treatment [6–11]. This distinction is important because it gives rise to different diagnostic approaches and epidemiological and preventive actions, particularly in countries where tuberculosis is endemic and infection by *M. bovis *continues to be identified. Infection by *M. bovis *requires intervention at the level of agricultural and food handling services to disrupt transmission to humans, whereas *M. tuberculosis *infection demands strengthening of public health measures to contain human-to-human spread [2]. 

Intestinal tuberculosis can be primary or secondary. Primary intestinal tuberculosis is caused by *M. bovis *and results from the ingestion of contaminated milk from infected cows. Secondary intestinal tuberculosis originates in an extraintestinal primary focus, usually pulmonary, and extends to the intestinal tract either as a consequence of hematogenous dissemination in the form of miliary tuberculosis or following ingestion of contaminated pulmonary secretions. It is usually caused by *M. tuberculosis*.

Peritoneal tuberculosis, with no gastrointestinal (or genital) lesions, is, for practical purposes, always secondary and results from reactivation of a latent peritoneal focus from previous hematogenous spread or as a part of active pulmonary tuberculosis with miliary dissemination. It is usually caused by *M. tuberculosis.*

In recent years, there has been a renewed interest in extrapulmonary forms of tuberculosis as a complication of immunodeficiency in adults. In contrast, the experience in children is meager, and most reports refer to isolated cases [12–15]. Moreover, there is very little information derived from postmortem studies [16]. Autopsy, in association with bacteriological support, is a powerful tool capable to identify and separate these forms of abdominal tuberculosis. 

The purposes of this review of our experience on autopsies of children with intestinal tuberculosis were

1. to determine the frequency of intestinal and of peritoneal involvement in patients dying with tuberculosis in whom an autopsy was performed,
2. to define the clinical and pathological features which distinguish primary from secondary intestinal tuberculosis and both from isolated peritoneal tuberculosis.

## 2. Material and Methods 

Cases material was drawn from the autopsy files of the Department of Pathology at the National Institute of Pediatrics in Mexico City. This is a referral institution and receives patients countrywide. From a total number of 7240 autopsies collected between 1971 and 2010, the autopsy records of 153 pediatric patients with tuberculosis were reviewed, and those with gross and microscopic features of intestinal or peritoneal involvement were selected. In the histologic study of the intestine and/or peritoneum, all these cases had granulomatous inflammatory process with caseating necrosis in which fungi were ruled out by special stains. 

Histological or bacteriological demonstration of acid fast bacilli was obtained in most cases either during life or at autopsy. From the clinical records, we retrieved information on age, sex, clinical manifestations, contact with tuberculous patients, duration of abdominal symptoms, and clinical diagnosis. 

From the autopsy report, we obtained the gross appearance and topography of the intestinal lesions. The slides were reviewed to confirm the tuberculous nature of the lesions. Results of bacterial studies were obtained from culture of involved tissues sampled at autopsy or during life. Differentiation of *M. tuberculosis *and *M. bovis *was carried out by the niacin accumulation and nitrate reduction tests [16]. The cases were classified as follows.

**Intestinal/Peritoneal Tuberculosis**

Cases in which signs, symptoms, clinical approach, and therapeutic decisions were predominantly related to abdominal disease.

**Isolated Peritoneal Tuberculosis**

Peritoneal involvement without intestinal lesions.

**Primary Intestinal Tuberculosis**

Involvement of the intestinal wall/mucosa with or without peritoneal lesions and with no evidence of primary pulmonary disease.

**Secondary Intestinal Tuberculosis**

Cases with progressive primary pulmonary tuberculosis and with intestinal involvement with or without peritoneal lesions as described previously.

## 3. Results 

### 3.1. Frequency (Figure 1)

Of the 153 cases of tuberculosis in our autopsy series, 24 (15.6%) patients had intestinal or peritoneal tuberculous disease. All of them had clinical manifestations consistent with the disease. Intestinal disease was present in 17 (71%), 14 of them with peritoneal involvement. Seven cases (29%) had peritoneal tuberculosis without presence of intestinal disease (isolated peritoneal disease). Most cases occurred before the year 2000, and none of our cases was associated with AIDS [17].

### 3.2. General Features of Peritoneal Tuberculosis (Table 1)  (Figure 2)

Most of the isolated peritoneal tuberculosis cases occurred as a result of blood borne spread from a primary infection elsewhere. A primary infection in the lung was present in four out of 7 cases. In two patients, calcified abdominal lymph nodes were present; one of them grew *M. bovis *which suggests a primary intestinal infection which had resolved. In one further case, no evidence of primary disease was identified despite a thorough search in thoracic and abdominal organs and in the genital tract. In none of the 7 cases was a human TB contact identified. All seven patients presented clinically with ascites and abdominal pain.

### 3.3. General Features of Intestinal Tuberculosis (Table 2)

Our cases of intestinal tuberculosis include 8 patients with primary intestinal tuberculosis and 9 with secondary intestinal tuberculosis. In 14 of these 17 cases, the intestinal lesion extended to the peritoneum with adhesions between bowel loops and with ascites in some cases. Secondary intestinal tuberculosis coexisted with active pulmonary tuberculosis. Most cases with intestinal tuberculosis, both primary and secondary, occurred in school age children. However, one half of the children with the primary form were under 2 years of age, whereas the other half of the children with the secondary form were over 10 years of age. 

*M. bovis *occurred only in the primary form of intestinal tuberculosis and was identified in 6 of our 8 patients (one of them was not cultured), and *M. tuberculosis *was found exclusively in secondary intestinal tuberculosis. As expected, children with secondary intestinal tuberculosis and with active pulmonary disease had an identified human TB contact, usually in the immediate family, whereas in the primary form such contact was documented in only one case. 

A correct clinical diagnosis of intestinal tuberculoses was achieved in all our primary cases but in only 5 of the 9 cases with the secondary form. In general, the cases with secondary forms were not seen by gastroenterologists, and the necessary diagnostic procedures were not considered. In contrast, in three of our primary cases, exploratory laparotomies were performed and biopsies taken. The main gastrointestinal clinical manifestations were abdominal pain (100%), diarrhea (76%), ascites (70%), and a palpable abdominal mass (59%). Gastrointestinal tract bleeding appeared more often in the secondary form and intestinal obstruction in the primary form. 

### 3.4. Pathological Findings (Table 3)   (Figures 3–5)

**Figure 3:**
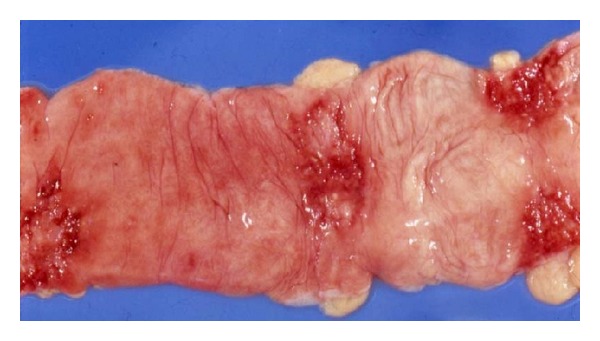
Intestinal tuberculosis. Ulcer with irregular, indurated borders in ileum.

**Figure 4:**
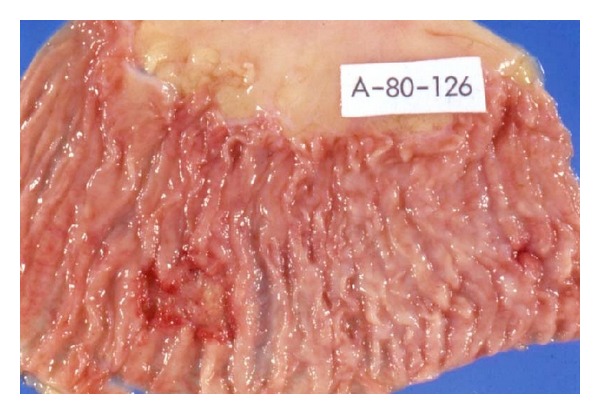
Intestinal tuberculosis. Irregular transverse ulcers in colon.

**Figure 5:**
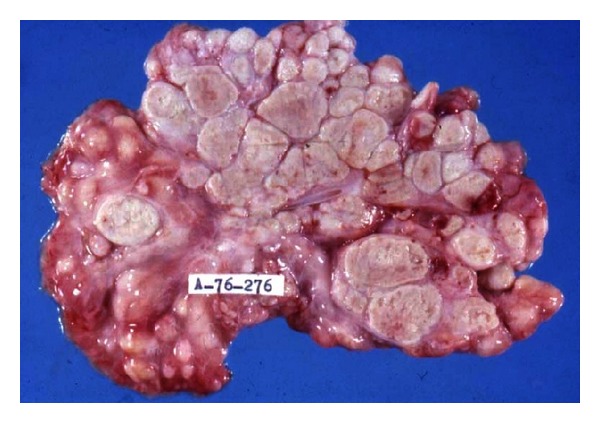
Mesenteric tuberculosis. Massive involvement of mesenteric lymph nodes with extensive caseation.

In both the primary and secondary forms, the intestinal lesions consisted in ulcers and affected mainly the ileum (13/17) (Figure 3) and colon (9/17) (Figure 4). Extension to peritoneal disease was present in 14 of our 17 cases. Regardless of the site of primary infection, all cases had involvement of mesenteric lymph nodes with extensive caseation (Figure 5). In contrast, mediastinal node involvement was present in all cases of secondary intestinal tuberculosis with an active primary lung lesion and in only one half of those with primary intestinal tuberculosis. Dissemination was more extensive in secondary disease as compared with primary intestinal tuberculosis. It is noteworthy that central nervous system involvement was more common in primary intestinal tuberculosis and was present in 3 of 6 cases of intestinal tuberculosis in which *M. bovis *was isolated.

## 4. Discussion 

The frequency of intestinal tuberculosis in autopsy material varies among different series. A recent review by Sharma and Bhatia reported a frequency of 55%–90% of cases with tuberculosis, but the characteristics of the populations analyzed are not specified [9]. It is important to emphasize that children with tuberculosis die with widely disseminated disease, and granulomas are found at autopsy in many organs including the intestinal tract and peritoneum, liver, spleen, kidneys, and bone marrow. In this study, we concentrated on abdominal disease. We selected cases with intestinal/peritoneal involvement, and these cases presented with a consistent clinical picture, which may or may not have been detected during the hospital course of the patient but which retrospectively fulfilled the criteria of abdominal disease. 

Peritoneal/intestinal disease occurred in 15.6% of the cases dying from tuberculosis over a 39-year period; it is second in frequency after pulmonary disease. This high frequency contrasts with the common appreciation that this is a very infrequent complication of tuberculous infections in childhood, possibly explained by its difficult diagnosis in the living patient [14, 15]. As it has been stated in the literature, this is a disease of school age children, adolescents, and young adults [9, 16]. One half of our cases are below 6 years of age with a chronic clinical course of an average of 6-months duration. A clear predominance in girls agrees with the experience of several Indian studies [6, 9]. 

Notable differences between primary and secondary intestinal tuberculosis can be summarized as follows.

1. *M. bovis *was the most frequent etiologic agent in primary intestinal tuberculosis and exclusive to this form, which indicates that the ingestion of milk from diseased cows is the main pathogenic mechanism [2, 5]. The secondary form, on the other hand, may result from hematogenous seeding or from the ingestion of contaminated material from pulmonary lesions in patients infected with *M. tuberculosis* [18].
2. Children with primary intestinal tuberculosis die at a younger age than those with the secondary form in spite of a prolonged clinical course. This suggests, as our data shows, that primary infection with *M. bovis *occurred at an earlier age.
3. As it would be expected, children with secondary intestinal tuberculosis frequently (4/9; 44.4%) had human TB contacts identified usually in the immediate family, whereas in the primary form a contact was documented in only one (12.5%) of eight cases.
4. The clinical diagnosis in the secondary form is difficult and requires a high degree of suspicion in order to detect an abdominal complication in a patient with pulmonary disease.
5. Children dying from primary intestinal tuberculosis tended to present more frequently with obstruction, whereas those with secondary intestinal tuberculosis presented with gastrointestinal hemorrhage possibly related to systemic disease widely disseminated in liver, spleen, and bone marrow, with disturbed hemostasis.
6. Although histological evidence of lympho-hematogenous dissemination occurred in both primary and secondary forms, central nervous system involvement was more frequent in infection by *M. bovis. *We have no explanation for this finding.
7. In our series, twenty (95.2%) of 21 children with evidence of peritoneal tuberculosis at autopsy had evidence of disease arising from a site other than the peritoneum, either secondary to a pulmonary lesion or as an extension to the peritoneum from primary or secondary intestinal lesions. This would suggest that primary peritoneal tuberculosis is a rare clinical entity, and its use should be limited to those extremely unusual cases of direct peritoneal contamination during dialysis [14, 15]. The presence of ascites is a constant indicator of peritoneal disease, and the cases referred to in the literature of “dry” or “sclerotic” peritoneal tuberculosis are very seldom seen in children [12].

## 5. Conclusions 

Autopsy offers an expanded view of the extent of tubercular disease which goes beyond the reach of the clinical perspective. In the present approach to abdominal tuberculosis, postmortem analysis was able to differentiate primary from secondary intestinal tuberculoses and to define the nature of peritoneal involvement by the disease. The feedback from this source of information strengthens the clinical insight on the diversity of tuberculosis.

## Figures and Tables

**Figure 1:**
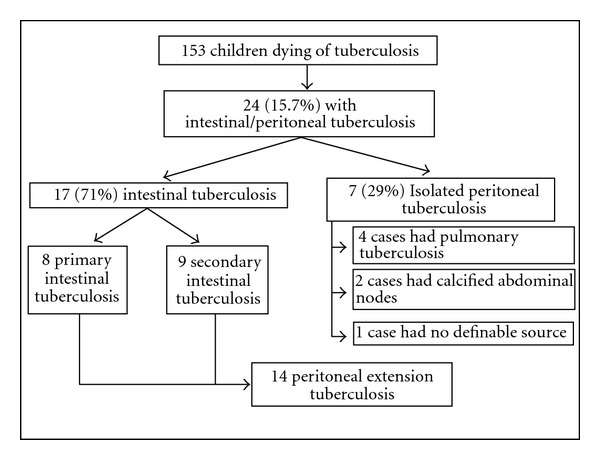
General classification of autopsy material.

**Figure 2:**
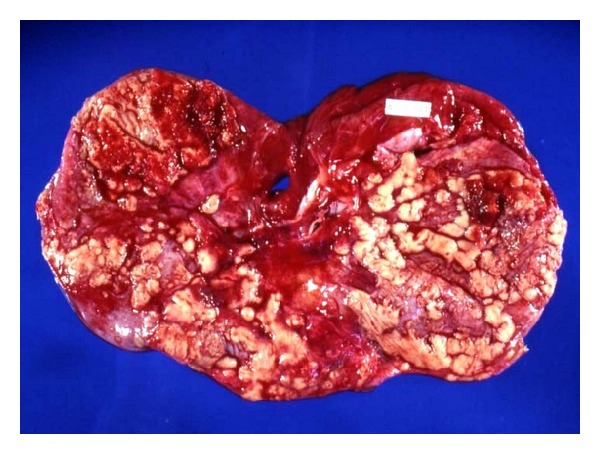
Peritoneal tuberculosis. Numerous granulomas on the abdominal aspect of the diaphragm.

**Table 1:** Isolated peritoneal tuberculosis: general features (7 cases).

Age (months)	
Range (min–max)	9–168
Mean	92
Median	84
Duration (months)	
Range (min–max)	1–6
Mean	3.8
Median	4
Gender	
Female	1
Male	6
Human TB contact	0
Etiology	
*M. tuberculosis *	2
*M. bovis *	1
Negative	4
Primary infection	
Pulmonary	4
Probably intestinal	2
Not determined	1
Dissemination	
Generalized (miliary)	5
Localized (peritoneal)	2

**Table 2:** Intestinal tuberculosis: general features (17 cases).

	Primary 8 cases	Secondary 9 cases	Total 17 cases
Age (months)			
Range	16–152	5–156	5–156
Mean	67	92	80
Median	48	120	72
Duration (months)			
Range	3–36	0.5–12	0.5–36
Mean	9.8	5.3	7.5
Median	6	6	6
Gender			
Female	6	6	12
Male	2	3	5
Human TB contact	1/8	4/9	5/17
Etiology*			
M. bovis*	6	0	6
*M. tuberculosis *	0	2	2
M. unclassified	1	1	2
Negative	0	3	3
Not cultured	1	3	4
Clinical diagnosis			
Tuberculosis	8	5	13
Gastroenteritis	0	3	3
Lymphoma	0	1	1
Local symptoms			
Abdominal pain	8 (47%)	9 (53%)	17
Diarrhea	6 (46%)	7 (54%)	13
Ascites	5 (42%)	7 (58%)	12
Abdominal mass	4 (40%)	6 (60%)	10
Perforation	3 (43%)	4 (57%)	7
Bleeding	1 (20%)	4 (80%)	5
Intestinal obstruction	3 (75%)	1 (25%)	4

*Statistically significant, *P* = 0.02, Fisher's exact test.

**Table 3:** Intestinal tuberculosis: pathological features.

	Primary 8 cases	Secondary 9 cases	Total 17 cases
Lesions			
Peritonitis	7	7	14
Ulcers, ileocolic	4	4	8
Ulcers, jejunum/ileum	2	3	5
Ulcers, colon	1	0	1
Dissemination			
Mesenteric lymph nodes	8 (47%)	9 (53%)	17
Liver	7 (47%)	8 (53%)	15
Lung	6 (40%)	9 (60%)	15
Spleen	8 (53%)	7 (47%)	15
Mediastinal lymph nodes*	4 (31%)	9 (69%)	13
Kidney	3 (43%)	4 (57%)	7
Bone marrow	2 (29%)	5 (71%)	7
Central nervous system	4 (67%)	2 (33%)	6
Pancreas	1	2	3
Pleura	2	0	2
Thoracic wall	2	0	2
Adrenal	0	2	2
Larynx	0	2	2
Tonsils	0	1	1
Bladder	0	1	1
Uterus	0	1	1
Pericardium	0	1	1

*Statistically significant, *P* = 0.02, Fisher's exact test.
